# ISG20: an enigmatic antiviral RNase targeting multiple viruses

**DOI:** 10.1002/2211-5463.13382

**Published:** 2022-02-27

**Authors:** Séverine Deymier, Camille Louvat, Francesca Fiorini, Andrea Cimarelli

**Affiliations:** ^1^ Centre International de Recherche en Infectiologie (CIRI) Université de Lyon Inserm U1111 Université Claude Bernard Lyon 1 CNRS UMR5308 École Nationale Supérieur de Lyon France; ^2^ UMR 5086 ‐ CNRS / UCBL IBCP Lyon France

**Keywords:** epitranscriptomic modifications, interferon, ISG20, RNase, translational inhibition, virus inhibition

## Abstract

Interferon‐stimulated gene 20 kDa protein (ISG20) is a relatively understudied antiviral protein capable of inhibiting a broad spectrum of viruses. ISG20 exhibits strong RNase properties, and it belongs to the large family of DEDD exonucleases, present in both prokaryotes and eukaryotes. ISG20 was initially characterized as having strong RNase activity *in vitro*, suggesting that its inhibitory effects are mediated via direct degradation of viral RNAs. This mechanism of action has since been further elucidated and additional antiviral activities of ISG20 highlighted, including direct degradation of deaminated viral DNA and translational inhibition of viral RNA and nonself RNAs. This review focuses on the current understanding of the main molecular mechanisms of viral inhibition by ISG20 and discusses the latest developments on the features that govern specificity or resistance to its action.

## Graphical abstract

ISG20 is a broad antiviral inhibitor and a potent RNase *in vitro*. However, a precise understanding of its mechanism of action is lacking. In this review, we discuss the latest developments on this fascinating innate defense factor.

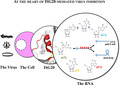

**Abbreviations**
+ssRNA: positive‐strand RNA
AEN: apoptosis‐enhancing nuclease
APOBEC3A: apolipoprotein B mRNA‐editing enzyme catalytic subunit 3A
CB: cajal bodies
cccDNA: covalently closed circular DNA
CHIKV: chikungunya virus
CrPV: cricket paralysis virus
DENV: dengue virus
dsRNA/DNA: double‐stranded RNA or DNA
E box: enhancer box
eif2: eukaryotic initiation factor 2
eif4: eukaryotic initiation factor 4
eIF4F: eukaryotic initiation factor 4F
Exo: exonuclease motif
HBV: hepatitis B virus
HCV: hepatitis C virus
HEM45: human estrogen‐regulated transcript 45 protein
HIV‐1: human immunodeficiency virus type 1
I: inosine
IAV: influenza A virus
IFIT1: interferon‐tetrapeptide repeat protein 1
IFN: interferon
IFNAR1/2: interferon alpha and beta receptor subunit 1 or 2
IFN‐I (IFNα/β): type I interferon
IFN‐II (IFN gamma): type II interferon
IRES: internal ribosome entry site
IRF1/3/7/9: interferon regulatory factor 1, 3, 7, or 9
ISG: interferon‐stimulated gene
ISG20: interferon‐stimulated gene 20kDa protein
ISG20L1: interferon‐stimulated exonuclease gene 20kDa‐like 1
ISG20L2: interferon‐stimulated exonuclease gene 20kDa‐like 2
ISRE: interferon‐stimulated response element
JAK‐STAT: Janus kinase signal transducer and activator of transcription
m1A: N1‐methyladenosine
m5C: 5‐methylcytosine
m6A: N6‐methyladenosine modification
mISG20: mouse ISG20
mTOR: mammalian target of rapamycin
NP: nucleoprotein
NS: nonstructural
P bodies: processing bodies
PAMP: pathogen*‐*associated molecular pattern
pgRNA: pregenomic RNA
Poly(I:C): polyinosinic:polycytidylic acid
PRR: pattern recognition receptor
rDNA: relaxed circular DNA
RdRp: RNA‐dependent RNA polymerase
RIG‐I: retinoic acid‐inducible gene I
RNaseL: ribonuclease L (for latent)
RT: reverse transcriptase
SARS‐CoV: severe acute respiratory syndrome coronavirus
snoRNA: small nucleolar RNA
snRNA: small nuclear RNA
Sp‐1: specificity protein 1
‐ssRNA: negative‐strand RNA
ssRNA/DNA: single‐stranded RNA or DNA
STAT1/2: signal transducer and activator of transcription 1 or 2
TLR3: Toll‐like receptor 3
UMP: uridine 5'‐monophosphate
USF‐1: upstream stimulatory factor 1
VEEV: Venezuelan equine encephalitis virus
VSV: vesicular stomatitis virus
WNV: West Nile virus
WT: wild‐type
YTHDF2: YTH‐domain family 2
ε: epsilon
ψ: pseudouridine

## A general canvas for the cellular context of expression of ISG20

ISG20 is an exquisite interferon‐regulated protein, and as such, what follows is a very general introduction to interferons (IFNs). The interferon system represents the first line of response against microbial infections [1, 2, 3]. During infection, the cell recognizes pathogen‐associated molecular patterns (PAMPs) via pattern recognition receptors (PRRs). Recognition initiates signaling cascades that converge onto several transcription factors (IRF3, IRF7, NF‐κB…) that migrate in the nucleus and lead to the synthesis of type I interferons (IFN‐I; IFNα/β). Contrary to more specialized interferons (i.e., types II and III, which are specific to immune cells), IFN‐Is are instrumental to induce a generalized antiviral state and to start innate immune responses.

In humans, the IFN locus on human chromosome 9p contains 13 functional α genes *(IFNα1, IFNα2, IFNα4, IFNα5, IFNα6, IFNα7, IFNα8, IFNα10, IFNα13, IFNα14, IFNα16, IFNα17,* and *IFNα21)*, one β gene situated syntenically (IFNβ1) [4, 5], and three more distantly related IFN genes (*IFNε, IFNκ*, and *IFNω*). Members of the IFN family exhibit from 30 to 80% sequence identity, and all are ligands for the cell surface receptors IFNα receptor subunits 1 and 2 (IFNAR1/2). The ligand–receptor interaction leads to the activation of the Janus kinase signal transducer and activator of transcription (JAK‐STAT) signaling pathway and to the upregulation of several hundreds of genes, referred to as interferon‐stimulated genes (ISGs). These genes exert numerous effects aimed at blocking the microbe at multiple steps (entry into the cell, RNA processing, translation, etc.) [4, 5, 6].

In a very simplified manner then, this innate defense mechanism recognizes common features present in a given class of pathogens (reverse transcription as a blueprint for retroviruses, lipopolysaccharide for bacteria, etc.) in cells that are compromised. Through the rapid diffusion of signals (the IFNs), this leads to a generalized antimicrobial cellular state in which cells become more resistant to infection. This intrinsic cellular resistance is ultimately mediated by interferon‐sensitive genes (ISGs) that act at multiple layers to inhibit pathogen replication either by directly targeting it or by indirectly affecting cellular processes normally required for its replication.

If the mechanism of action of some ISGs is well described in the literature, the role of many others remains either unknown or unclear.

Among ISGs that act as antiviral effectors and that are thus thought to inhibit virus infection by directly binding or altering one of their components is the interferon‐stimulated gene 20 kDa protein (ISG20), also known as human estrogen‐regulated transcript 45 protein (HEM45) [7, 8]. ISG20 is the second RNase described to be induced by the IFN system after RNase L [9]. Despite the fact that initial studies reported a simple and direct explanation for viral inhibition by ISG20 (i.e., ISG20 directly degrades viral RNA thanks to its RNase enzymatic properties) [10], the following studies have somewhat complexified our view of how ISG20 may act. The following paragraphs will specifically focus on the discovery of ISG20, on the mechanisms that over the years have been ascribed to ISG20 and to open questions that may help decorticate the antiviral functions of this broad‐spectrum antiviral factor.

## A historical overview of the discovery of ISG20

ISG20 was discovered in 1997 in Daudi cells as a new interferon‐regulated protein [7]. Later studies demonstrated that ISG20 could be induced transcriptionally by both type I (α/β) and type II (γ) IFN through an unique interferon‐stimulated response element (ISRE) present in its promoter region, under the control of the IFN regulatory factor 1 (IRF1) [11, 12, 13]. Despite being clearly upregulated by IFNs, ISG20 exhibits also variable expression levels at the basal state in several cell types under the control of different transcription factor, as the specificity protein 1 (SP‐1) or the upstream stimulatory factor 1 (USF‐1), so that ISG20 could have a physiological impact on cellular functions also in the absence of ongoing interferon responses [11]. Independently from the study of Gongora et al, ISG20 was identified as a human estrogen‐regulated transcript (HEM45) in breast cancer cell lines [8], hence its double name ISG20/HEM45. Several reports suggest a link between the tumorigenic process and ISG20 [14, 15, 16, 17], although the exact mechanism through which ISG20 could influence this process remains unclear [18, 19, 20].

**Fig. 1:**
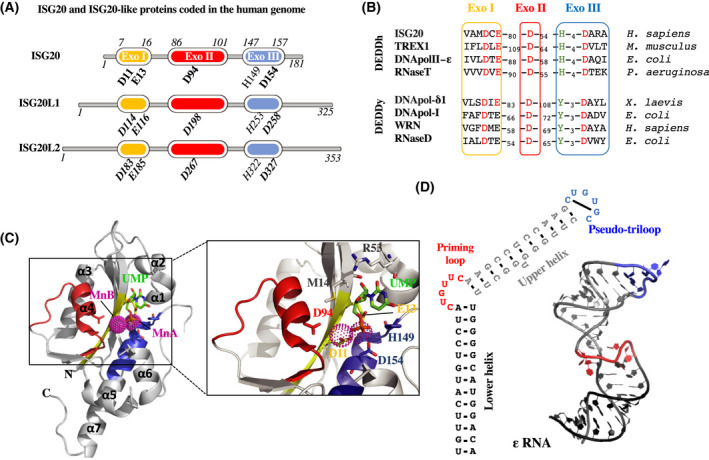
ISG20 belongs to the conserved group of DEDDh exonuclease. (A) Schematic structures of ISG20 and its human homologs. Critical DEDD residues and the h amino acid that defines the proteins’ nucleic acid specificity are indicated (bold and not bold, respectively, with relative amino acid position on protein). (B) Alignment of a selection of proteins belonging to the DEDDy or DEDDh groups of the DEDD superfamily. DEDD residues are shown in red, while specific h or y amino acids are shown in green. (C) Crystal structure of human ISG20 in complex with two manganese (Mn) ions and uridine 5’‐monophosphate (UMP) ([29]; PDB:1WLJ). ExoI, ExoII, and ExoIII domains are respectively in yellow, red, and blue. UMP is in stick representation in green, and Mn ions are depicted as pink spheres. The inset on the left shows a stereo view of the interaction of UMP and the two Mn ions with the residues of the active site represented in sticks. (D) The secondary structure of HBV ε RNA: The basal stem is in black, the following bulge in red, the upper stem in gray, and the terminal hexaloop in blue. On the left is depicted the NMD structure of the ε RNA that has been recently solved ([34]; PDB:6VAR).

In humans, *isg20* is located on the chromosome 15q26.1 and codes for a 181 amino acid protein [10, 21]. Humans code for two additional proteins bearing high homology with ISG20: the interferon‐stimulated exonuclease gene 20 kDa‐like 1 and 2 (ISG20L1 and ISG20L2, which share 53 and 48% identity with ISG20, respectively) (Fig. 1A). *isg20l1* lays just next to *isg20* and has been involved in apoptosis, hence its name of apoptosis‐enhancing nuclease (AEN), while *isg20l2* is located on chromosome 1q23.1 and has been involved in ribosome biogenesis. At this time, neither ISG20L1 nor ISG20L2 has been reported to possess antiviral activities [22, 23, 24].

## ISG20, a member of the DnaQ‐like (or DEDD) 3'‐5' exonuclease domain superfamily

The DEDD superfamily of 3'‐exonucleases includes a large number of prokaryotic and eukaryotic enzymes that cleave one at a time from the 3’‐RNA and/or DNA terminus [25]. Exoribonucleases of this family can be autonomous enzymes or integrated into larger replicative complexes, supporting, for example, the proofreading functions of DNA polymerases, of DNA repair enzymes, or of RNA maturation and turnover complexes [26]. The architecture of DEDD exonucleases is as conserved as their mechanism of catalysis: The catalytic site is composed of four conserved acidic amino acid residues (Asp‐Glu‐Asp‐Asp) distributed within three exonuclease motifs named ExoI, ExoII, and ExoIII [27, 28]. These residues coordinate the two metal ions responsible for the hydrolysis of the last phosphodiester bond of RNA or DNA. Furthermore, the superfamily is subdivided into two major groups, DEDDh and DEDDy, according to the presence of a fifth highly conserved residue within the ExoIII motif, which can be either a histidine (H) or tyrosine (Y) [27] (Fig. 1B). The role of these alternative residues in catalytic activity or substrate binding is still unclear.

ISG20 belongs to the DEDDh subgroup of 3’ to 5’ exonucleases as a large number of independent RNases such as the ribonuclease T and D, the three prime repair exonuclease 1 (TREX1), or the yeast RNA exonuclease 4 homolog (REX4) (Fig. 1B). Because of the conservation of their basic fold and active site, DEDDh exonucleases have divergent substrate‐binding sites due to their different substrate preferences. ISG20 degrades single‐stranded RNA (ssRNA) and DNA (ssDNA) *in vitro* with a strong preference for RNA [10]. The crystal structure of ISG20 complexed with two manganese (Mn) ions and uridine 5’‐monophosphate (UMP) has been solved at 1.9 Å of resolution [29](PDB:1WLJ). In this structure, a five‐stranded β‐sheet core is surrounded by two clusters of α‐helices: α3, α4 and α1, α2, α5, α7 (Fig. 1C). Within the active site, the Asp11, Glu13, and Asp154 coordinate MnA, while MnB is coordinated directly by Asp11 and indirectly through water molecules by Asp94 (Fig. 1C, adapted from Horio et al. [29], PDB:1WLJ). Only the Mn^2+^ showed stimulatory effects on ISG20 activity, whereas other bivalent cations such Mg^2+^, Ca^2+^, or Zn^2+^ did not [10]. Importantly, the conserved His149 interacts with the O2P of the UMP phosphate group, suggesting that this residue cannot be responsible for the preference shown by ISG20 for ssRNA to ssDNA [10, 29]. This preference might be exerted by Met14 and Arg53 (Fig. 1C), because they directly interact with the ribose 2′‐OH group of the UMP [10, 29]. However, deeper mutational analyses or the structure of RNA‐bound ISG20 are needed to better understand the molecular bases of ISG20 preference for RNA substrates. Furthermore, concerning the enzymatic mechanism, whether ISG20 acts as a processive or a distributive enzyme has also not been properly investigated so far.

While the purified ISG20, free from any cellular interactor, cleaves essentially ssRNA *in vitro*, the 3’‐structured substrates are likely resistant [10, 30]. This resistance has been well characterized in the case of the hepatitis B virus (HBV) RNA encapsidation signal called epsilon (ε) [30, 31]. The ε RNA is located near the 5' end of the HBV pregenomic RNA, and it exhibits a long 13‐bp basal stem followed by a 6‐nt bulge region also called priming loop, a 11‐bp upper stem and a terminal hexaloop [32, 33] (Fig. 1D). The first full‐length structure of HBV ε RNA has been recently solved using solution nuclear magnetic resonance (NMR) spectroscopy [34] (PDB:6VAR; Fig. 1D). This structure is of interest in light of the fact that the specific binding of ISG20 to the HBV ε RNA takes place at the level of the lower stem, in a manner independent of the bulge sequence but dependent of the stem length. More than nine RNA base pairs of ε lower stem seem to be necessary for stable ISG20:ε interaction. Despite this efficient binding, ISG20 was not able to cleave ε RNA substrate [30]. Interestingly, a C‐terminal truncated version of ISG20, with the ExoIII motif deleted, has been shown to abolish RNA binding [30]. This suggests that ExoIII could harbor the RNA‐binding domain or, alternatively, that the coordination of the MnA by Asp154 is critic for RNA interaction (Fig. 1C).

Recently, a strong exonuclease activity of ISG20 has been reported on deoxyuridine‐containing ssDNA. This finding is of interest because deoxyuridine‐containing DNA is the result of the editing activity of a second important antiviral factor, the human apolipoprotein B mRNA‐editing catalytic polypeptide‐like 3A protein (APOBEC3A; in this case, the hepatitis B virus, HBV, and more generally of all members of this family) that deaminates cytosines on viral genomes [35]. This activity is of interest because it connects the concerted action of two antiviral factors in the mutagenesis and degradation of viral genomes, as we will discuss more extensively below.

## Virus inhibition by ISG20

In light of its exquisite activity against RNA, it is perhaps not surprising, although simplistic, that ISG20 has been described to interfere with the replication of a large spectrum of RNA viruses. This list ranges from negative‐ or positive‐strand RNA viruses such as *Rhabdoviridae* or *Flaviviridae* (for instance, the vesicular stomatitis virus, VSV, or the hepatitis C virus, HCV) to *Retroviridae* such as the human immunodeficiency type 1 virus (HIV‐1), the genome of which is reverse‐transcribed from a single‐stranded RNA to double‐stranded DNA [36, 37, 38, 39] (for an exhaustive list, Table 1).

**Table 1:** Virus susceptibility to ISG20 inhibition and known modifications of viral RNAs.

	ISG20 inhibition (Refs.)	Known viral RNA features			
		7mG cap (cap 1)	Epitranscr. Modif.	Poly(A) tail	Refs.
Negative‐polarity RNA viruses					
***Orthomyxoviridae*** Influenza A virus (IAV)	Yes; [37, 96]	Yes *Cap‐snatching*	m6A	Yes	[97, 98]
***Peribunyaviridae*** Bunyamwera (BUNV) Cache Valley virus (CVV) Kairi virus (KRIV) Oropouche virus (OROV) Schmallenberg virus (SBV) Batama virus (BMAV) Boraceia virus (BORV) Tacaiuma virus (TCMV) Anopheles A virus (ANAV) Capim virus (CAPV)	Yes; [80]	Yes *Cap‐snatching*	ND	No	[99, 100]
	No; [80]				
***Hantaviridae*** Puumala virus (PUUV)	Yes; [80]				
***Nairoviridae*** Dugbe virus (DUGV)					
***Phenuiviridae*** Rift Valley fever virus (RVFV) Severe fever with thrombocytopenia syndrome virus (SFTSV) Heartland virus (HRTV)	No; [80]				
***Rhabdoviridae*** Vesicular stomatitis virus (VSV)	Yes; [37, 39]	Yes	m6A	Yes	[101, 102, 103]
Positive‐polarity RNA viruses					
***Flaviviridae*** Hepatitis C virus (HCV) Bovine viral diarrhea virus (BVDV) West Nile virus (WNV) Dengue virus (DENV) Yellow fever virus (YFV) Zika virus (ZIKV)	Yes; [24, 104, 105]	No	m6A, m5C, ψ, m1A, I	No	[89, 106, 107, 108, 109]
	Yes; [24]		ND	No	[107, 110]
	Yes; [38]	Yes	m6A (WNV) m6A, m5C, ψ, m1A, I (DENV)	No	[89, 108, 111, 112, 113, 114, 115]
	Yes; [24]		m6A, I		
	Yes; [93]		m6A, m5C, ψ, m1A		
***Picornaviridae*** Hepatitis A virus HAV Encephalomyocarditis virus (ECMV)	Yes; [24, 37]	No	ND	Yes	[116, 117, 118]
***Togaviridae*** Sindbis virus (SINV) Chikungunya virus (CHIKV) Venezuelan equine encephalitis virus (VEEV)	Yes; [12, 119]	No *Cap* 0*	ND	Yes	[120, 121]
***Coronaviridae*** Severe acute respiratory syndrome coronavirus (SARS‐CoV‐1) and SARS‐CoV‐2	No (1); [24] Yes (2); [122]	Yes	ND m6A (SARS‐CoV‐2)	Yes	[123, 124, 125]
Viruses containing retroelements					
***Retroviridae*** Human immunodeficiency virus type 1 (HIV‐1)	Yes; [36, 126, 127]	Yes	m6A, m5C, ψ, m1A, I	Yes	[108, 128, 129, 130, 131]
***Hepadnaviridae*** Hepatitis B virus (HBV)	Yes; [21, 132, 133]	Yes	m6A	Yes	[50, 133, 134, 135]

Virus families are indicated in bold.

* Cap 0: m7GpppNp. Di‐ and trimethylated caps have also been described (m2,7G‐ and m2,2,7G‐caps in the case of SINV). ND, not determined.

**Fig. 2:**
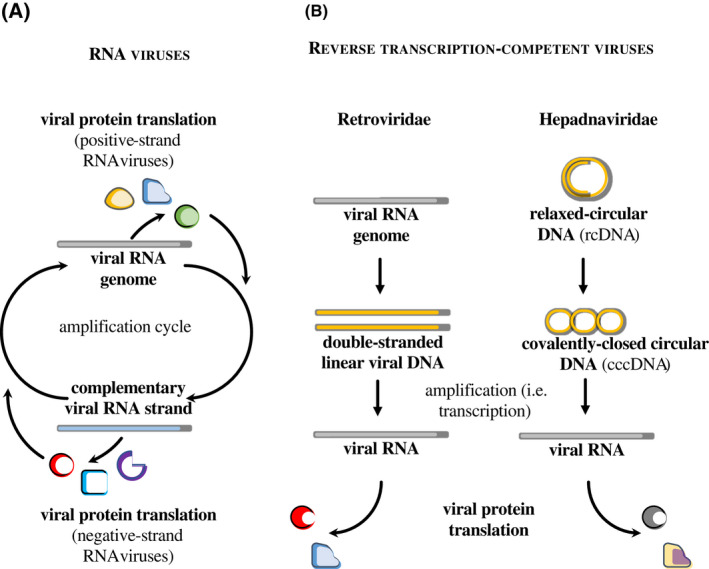
Strategies for viral genome amplification used by viruses reported to be inhibited by ISG20. (A) Simplified overview of the RNA amplification cycle used by positive‐ and negative‐strand RNA viruses. Upon entry, the RNA genome of positive‐strand RNA viruses is translated into viral proteins that promote synthesis of the complementary RNA strand and ensure the amplification cycle. In the case of the latter instead, a first round of pioneer RNA synthesis is needed to ensure the first round of viral protein translation. (B) Schematic overview of the strategy used by *Retroviridae* and *Hepadnaviridae* to replicate through an intermediate reverse transcription step. In this case, viral RNA is amplified through transcription of RNAs of positive polarity in the cell nucleus, a process that co‐opts the cellular RNA polymerase II.

From a very schematic manner, negative‐ and positive‐strand RNA viruses undergo an obligatory step of antigenomic RNA synthesis that leads to viral RNA amplification, translation of viral proteins, and ultimately the formation of novel virion particles (as schematically presented in Fig. 2A). The first round of viral translation, which is key to ensure the RNA amplification cycle, occurs either directly from the viral genome in the case of positive‐strand RNA viruses or after pioneer transcription in the case of the negative‐strand ones. The replication cycle of the viral genome is instead quite distinct in the case of *Retroviridae* and *Hepadnaviridae* that undergo a reverse transcription phase that leads to the formation of linear or circular DNA (Fig. 2B). These nucleic acids are first transported into the nucleus of host cells and then transcribed with the concourse of viral components, similar to cellular genes. In this case therefore, genome amplification is promoted by viral‐coded proteins, but is essentially assured by the cellular transcription machinery, and only RNAs of positive polarity are produced during infection [40, 41]. Of note, antigenome RNA transcription has been reported for some retroviruses, but the role of these transcripts is either unclear or transcriptional regulation [42, 43].

Even through the lenses of this simplified view of their mechanisms of replication, it is difficult to reconcile a single mechanism of inhibition based on the direct degradation of viral RNA against such diverse viruses. Indeed, while most RNA viruses targeted by ISG20 carry out their replication cycle entirely in the cell cytoplasm, some, as *Orthomyxoviridae*, replicate in the nucleus [44, 45, 46, 47]. Also replicating away from the cell cytoplasm are *Retroviridae* and *Hepadnaviridae,* the viral RNAs of which are transcribed in the cell nucleus as *bona fide* cellular mRNAs, endowed with a 5’ cap (7‐methylguanylate, m7G) and a 3’ poly A tail.

How can this enzyme distinguish between cellular and viral RNAs? One possible hypothesis would be that ISG20 is capable of discriminating RNAs based on specific features present, absent, or differentially present in one versus the other RNA species, as, for example, epitranscriptomic modifications, 5’ cap, or poly A tails. However, while some of the viruses targeted by ISG20 lack some of these canonical features present on cellular RNAs, other viruses do possess them (Table 1). As such, the exact mechanism through which ISG20 identifies its RNA targets remains unclear.

At present, the results obtained and confirmed by numerous laboratories concur in three experimental evidences: ISG20 behaves as a potent RNase *in vitro*; this enzyme inhibits the replication of numerous viruses and its enzymatic activity is key for this property, given that a single point mutation that disrupts the catalytic site of ISG20 both abolishes its ability to degrade RNA *in vitro* and impairs its antiviral behavior. The explanation of how this behavior underlines the antiviral mechanism of action of ISG20 is still a matter of debate, and currently, four models have been proposed: two in which ISG20 directly degrades its viral target and two in which ISG20 blocks the translation from viral RNA without degrading them. Given that the RNase activity of ISG20 remains nonetheless important for this translational block, it follows that ISG20 must target a cellular RNA, whose identity remains, however, unknown.

## Mechanism 1: direct viral RNA degradation

**Fig. 3:**
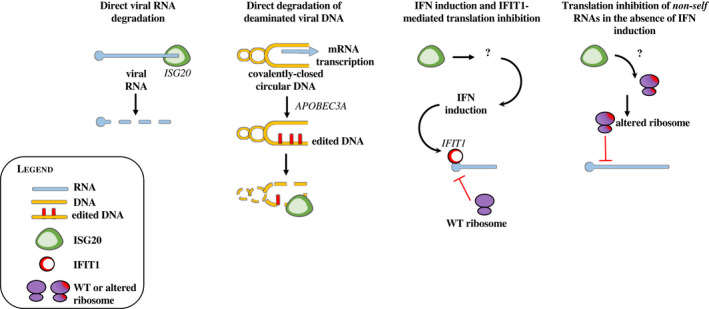
Proposed mechanisms of viral inhibition by ISG20. The figure presents the four models of action that have been ascribed to ISG20. From left to right, in the first two models ISG20 has been proposed to directly degrade viral nucleic acid in its RNA or deaminated DNA forms (models 1 and 2, respectively). In models 3 and 4, ISG20 has been proposed to inhibit viral translation but not to promote direct viral RNA degradation. In model 3, translation inhibition is promoted through an unknown mechanism by ISG20 by the induction of IFN and more specifically of IFIT1, a well‐known translation inhibitor that targets viral RNAs devoid of a proper 5’ cap. In model 4, translation inhibition occurs independently from IFN induction and results in a defect in translation efficiency, likely due to the direct modification of the translation machinery. Also, in this case, the identity of the targeted RNAs remains unknown.

The first model proposed on the basis of the experimental evidence described above was that ISG20 directly degraded viral RNA [30, 36, 37, 48, 49] (Fig. 3). Indeed, the expression of ISG20 led to lower accumulation of viral RNA and synthetic viral RNA mimics were also efficiently degraded *in vitro*.

In addition to the fact that the direct binding of WT ISG20 to viral RNA has not been formally demonstrated in cells, the major caveat of this model is that it fails to provide the basis through which ISG20 can distinguish viral from cellular RNA substrates. Indeed, ISG20 is capable of degrading most ssRNA *in vitro* [10, 29], not just viral substrates so that the question of how ISG20 spares cellular RNAs remains unaddressed. At present, a single study reported the specific binding of ISG20 to the adenosine‐methylated (m6A, adenosine in position 1907) form of the epsilon structure of the HBV RNA (ε), a particular stem–loop structure and unique site for m6A modification on the HBV transcripts (or two sites within two identical ε in 5’ or 3’ ends for the pregenomic RNA) [50]. However, the association between ISG20 and this methylated structure was mediated through a direct protein–protein interaction with the m6A reader YTH‐domain family 2 (YTHDF2). Given the extent of the m6A modifications on cellular mRNAs [51], it is difficult to envision how YTHDF2 binding, alone, could help ISG20 discriminate between cellular and viral mRNAs. However, this finding is of interest because it raises the possibility that ISG20 connects with the complex epitranscriptomic machinery, perhaps in combination with other RNA features as, for instance, the polyA tail or the 5’ cap. The heterogeneous modifications and variable presence of these modifications among the large panel of viruses targeted by ISG20 do not allow for the moment the identification of a unique feature driving susceptibility or resistance to ISG20.

## Mechanism number 2. Direct degradation of deaminated viral DNA

While the activity of ISG20 on ssDNA is generally lower when compared to ssRNA *in vitro*, a recent study indicated that it could be increased in the presence of deoxyuridines (dU) [35]. In so doing, the authors of the study proposed an interesting model of cooperativity between two cellular antiviral factors against HBV, connecting the cytosine deamination activity of the apolipoprotein B mRNA‐editing enzyme catalytic subunit 3A (APOBEC3A) to the ensuing degradative activity of ISG20.

Members of the APOBEC3 family (A through H in humans) are prototype antiviral factors well studied in the context of retroviral infection [52, 53]. In this case, APOBEC3s are incorporated into virion particles and act during the process of reverse transcription in the cytoplasm of target cells by deaminating cytosines in the first strand of reverse‐transcribed viral DNA (the negative‐strand). As a result, deaminated C > U sites are misread when this strand is used as a template for the synthesis of the second strand (the positive‐strand) resulting in G‐to‐A mutations along the viral genome. For this family of viruses, the extent of accumulation of deleterious mutations along the viral genome (stop codons, mutations with negative effect on the protein functionality, etc.) can be so considerable that viral replication is severely impaired in a mechanism that has been referred to as death by mutagenesis [53, 54].

The mechanism of cytosine deamination described here in the case of HBV is distinct from the one of *Retroviridae*: first because it occurs in the nucleus and is carried out by APOBEC3A, which is together with APOBEC3B, the sole nuclear APOBEC3 member; and second, because it is carried out on double‐stranded covalently closed circular DNA (cccDNA) [55]. Its outcome is therefore not the introduction of mutations in the viral genome, but rather the stamping of viral DNA for ISG20‐mediated degradation [35]. Given that APOBEC3 proteins require a single‐strand DNA intermediate to act [56, 57], it is likely that APOBEC3A acts on transcriptionally active cccDNA in which the transcription machinery opens up the double‐stranded DNA (Fig. 3).

As said before, this mechanism of action is of interest because it models the concerted action of two antiviral factors. Several questions remain, however, unaddressed around the specificity of action of ISG20. First, the major enzymatic functions of ISG20 are directed against RNA, and second, most DNA viruses do replicate in the nucleus, but for the most part seem resistant to ISG20 [37, 39]. It will be therefore important to understand how these viruses avoid the combined action of APOBEC3A‐ISG20. The restricted pattern of expression of APOBEC3A to cells of the myeloid lineage could provide a first line of specificity to the action of ISG20 [58], but certainly cannot explain it alone. Third and more importantly, the cellular genome is constantly exposed to spontaneous deamination [59, 60]. It will be thus imperative to determine how the cellular genome protects itself from ISG20.

## Mechanism 3: IFN induction and IFIT1‐mediated translation inhibition of viral RNA

For most highly replicating RNA viruses, the first round of translation of viral proteins is capital to ensure viral RNA amplification. As such, an alternative explanation for the lower accumulation of viral RNA observed in numerous studies in the presence of ISG20 could be an inhibitory effect of the first round of viral translation, which would severely undermine the extent of viral RNA amplification.

Translation inhibition has been reported in mouse embryonic fibroblasts (MEF) overexpressing the murine ISG20 (mISG20) that shares 82% identity with human ISG20 (Fig. 3). In this study and in agreement with other studies, the authors found that ISG20‐mediated inhibition of the chikungunya virus (CHIKV) or Venezuelan equine encephalitis virus (VEEV) did not involve the degradation of viral RNAs, but rather the inhibition of translation of viral proteins [12]. Interestingly, translation inhibition was indirectly due to the stimulation of IFN‐I responses and more specifically to the expression of the interferon‐induced protein with tetratricopeptide 1 (IFIT1), a well‐known translation suppressor protein that targets uncapped viral RNAs [12, 61, 62]. In this model therefore, ISG20 inhibits viral translation indirectly through the induction of an IFN response and IFIT1. Despite the fact that a later study failed to confirm IFN and IFIT1 induction by ISG20 [39], this study opens up the possibility that ISG20 can drive larger responses besides direct viral inhibition.

This model leaves for the moment unanswered two major questions. First, ISG20 is itself an IFN‐regulated protein so that its reported induction of IFN would necessarily occur in an environment already primed by IFN. As such, either ISG20 acts as a positive regulator to potentiate or sustain IFN responses over time (perhaps by processing viral RNAs as RNaseL), or this mechanism is restricted to cell types in which ISG20 expression is induced by other signals, as, for example, estrogens. Second, this model does not explain the broad mechanism of viral inhibition of ISG20, as the spectrum of viruses inhibited by ISG20 is larger than those targeted by IFIT1 and it extends to viruses with a cellular‐like m7G‐5’ cap. Importantly, given that the exonuclease activity of ISG20 is important for this behavior, the identity of the RNAs (cellular or viral) targeted by ISG20 to induce IFN responses remains to be identified.

Mechanism 4: Translation inhibition of nonself RNAs in the absence of IFN induction. This mechanism was proposed recently by our laboratory following experimental observations that ISG20 exerted a strong impairment in the translation of viral proteins following VSV infection in spite of a very mild decrease in viral RNAs (Fig. 3) and in the absence of IFN‐I or IFIT1 induction [39].

Interestingly, a similar behavior could be reproduced upon ectopic transfection of plasmid DNAs independently from the nature of the reporter gene examined (i.e., cellular or viral, spliced or unspliced). On the contrary, ISG20 had no effect when this exact reporter cassette was stably integrated into the host genome.

Translation inhibition was independent from initiation factors as it affected equally reporter genes under the control of the cricket paralysis virus (*CrPV*) 5’‐untranslated region that drives initiation of factorless translation [63]. Interestingly, the suppressive effects of ISG20 were accompanied by its partial recruitment to cytoplasmic processing bodies (P bodies), important and dynamic sites of RNA metabolism [64], although it was not possible to determine whether this association was causal or secondary to the translational block itself [39].

VSV infection and transient DNA transfection do not share many features in common. However, they both represent the massive flooding of the cell with *nonself* genetic material, and in both cases, the process of cellular translation is largely redirected toward a single (in the case of ectopic DNA transfection) or a narrow set (in the case of viral infection) of RNA transcripts, a situation that is unusual for the cell and often associated with viral infection.

Given the absence of target RNA degradation, but in light of the importance of an intact RNase activity, we proposed that ISG20 could affect cellular translation by degrading a cellular RNA substrate, which affected proportionally the most actively translated RNAs, working as a threshold translational controller.

This model is of interest because it is relatively independent from a single RNA feature. However, it can be influenced by all of them and in particular by the extent of epitranscriptomic modifications that are added cotranscriptionally and thus depend on the chromatin status of the target gene.

In this respect, rRNAs and small nucleolar RNAs (rRNAs and snoRNAs, respectively) represent interesting potential targets with which ISG20 could alter the overall translational abilities of the cell. snoRNAs are small nonprotein coding RNAs (60‐300 nt) involved in a number of physiological processes among the post‐translational modification of rRNAs, process in which they guide the methylase fibrillarin on specific sites of rRNAs promoting 2’‐O‐methylation modifications [65, 66]. The weight of these modifications in the activity of ribosomes remains to be fully apprehended. However, an increasing number of reports support the notion that specialized subsets of ribosomes are formed in response to certain stimuli [67, 68, 69], and in this context, ISG20 could promote the formation of specialized pools of ribosomes, thus influencing the translational capacity of the cell.

This model would accommodate inhibition of a broad spectrum of viruses targeted by ISG20 according to a single mechanism of action. However, also in this case the identity of the RNAs targeted by ISG20 remains unidentified.

## Open question 1: the intracellular whereabouts of ISG20

The intracellular distribution of IGS20 has not been the subject of intense studies so far. Its small size (around 20 kDa) allows it to be fairly well distributed in both cell cytoplasm and nucleus. A subtler localization of ISG20 has been described upon strong detergent permeabilization in promyelocytic leukemia (PML) bodies, nucleolus, and Cajal bodies [7, 70]. PML bodies are involved in several physiological processes including antiviral responses, and they are often disrupted by viral infection [71, 72, 73]. Instead, nucleolus and CB are sites of formation and processing of rRNA and small nuclear ribonucleoproteins (snRNPs). An extensive literature exists on these discrete nuclear bodies, and the reader is invited to read some of the following reviews [74, 75, 76, 77, 78]. In light of the association of a fraction of ISG20 with these structures, it is thus possible that ISG20 is involved in small nuclear RNA (snRNA) and rRNA biogenesis and/or maturation [70], although this would support an indirect mechanism of viral inhibition by ISG20, rather than a direct one.

In the case of translation inhibition, our laboratory also reported that a fraction of cytoplasmic ISG20 could migrate (or induce) processing bodies (P bodies), which are cytoplasmic membraneless compartments involved in RNA decay and/or control of translation by RNA storage [39, 79], in line with their effect in translation inhibition.

However, the dynamics of ISG20 and an extensive characterization of its intracellular distribution during homeostasis or during the context of infection are cruelly lacking and this may yield important clues as to the mechanisms of inhibition of ISG20. For instance, with few exceptions [39], a single mutant is often used in several studies (D94, or similar mutations in the DEDD residues), but the use of additional mutants could extend our comprehension of the links between cellular localization of ISG20 and its antiviral activities. Similarly, although ISG20 is distributed in both cell cytoplasm and nucleus it is unclear whether trafficking between one and the other location is important for the antiviral activity. Certainly, this will be an interesting area of investigation for future studies.

## Open question 2: the specificity determinants of ISG20

While a large number of viruses are susceptible to the action of ISG20, several remain resistant to it. This includes not only DNA viruses such as adenoviruses or the Epstein–Barr virus but also RNA viruses such as several, although not all, members of *Phenuiviridae* [80], as well as SARS‐CoV [24]. *in vitro* ‐driven evolution toward resistance to ISG20 in Bunyamwera orthobunyavirus (BUNV), prototype virus of *Peribunyaviridae* family, has led to interesting hints about the determinants that may modulate susceptibility and resistance to this restriction factors [80]. Several mutations were indeed identified in BUNVs that adapted to replicate in the presence of ISG20 expression. These mutations occurred in the three segments of the viral genome (short (S), large (L), and medium (M)). Despite the fact that mutations in the different genome segments exhibited unequal contributions to the extent of viral replication likely due to their respective roles in this process, adaptive mutations were observed in potential structural RNA elements (mutations outside open reading frames, ORFs), as well as in the N and NSs proteins. Although the relative contribution of these mutations to ISG20 resistance has not been further dissected, these results indicate that both RNA‐binding proteins as the viral N protein and *cis*‐acting elements on the viral RNA may influence susceptibility to ISG20.

Viral proteins may indeed, as cellular proteins, shield viral RNAs from the degradative action of ISG20, and similarly, particular stem–loop structures may also protect RNA, as ISG20 displays no reported activity against double‐stranded RNA. In this respect, the RNA of bunyaviruses is not polyadenylated, but contains a conserved 3’ stem–loop structure that may be important to mediate the resistance of some, although not all, members of this viral order.

**Fig. 4:**
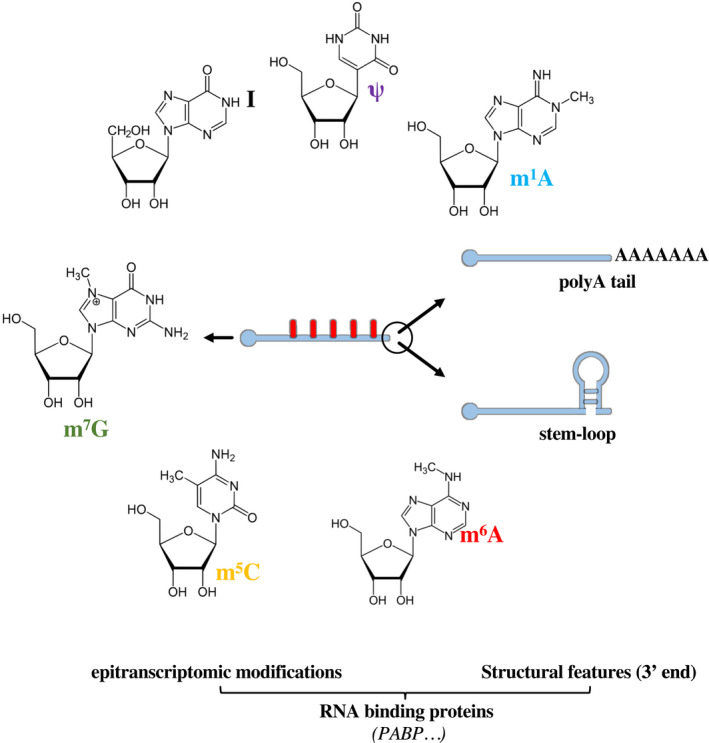
Features expected to modulate RNA susceptibility to ISG20. Intrinsic features of the RNA as poly A tail or secondary structures, as well as epitranscriptomic modifications that may in principle affect susceptibility to ISG20. The RNA‐binding proteins that can dock on the RNA as a consequence of these modifications are not shown for simplicity. m6A: N6‐methyladenosine, m5C: 5‐methylcytosine, m7G: N7‐methylguanosine, I: inosine, ψ: pseudouridine, m1A: N1‐methyladenosine.

In addition to the already‐mentioned 5’‐cap and poly(A) tail in the 3’ end, cellular mRNAs are also heavily modified either cotranscriptionally or shortly after transcription by the addition of the following modifications: ribose 2'‐O‐methylation, pseudouridine, inosine, m^6^A, and 5‐methylcytosine (Fig. 4). Collectively, these dynamic modifications are referred to as epitranscriptomic modifications and are involved in all aspects of the RNA biology, such as stability or efficiency of translation [81, 82], aspects that they influence either directly or indirectly through the recruitment of a large number of cellular proteins [83, 84]. Readers are invited to read the following reviews for a more complete overview of epitranscriptomic modifications [84, 85, 86, 87, 88].

These modifications are mentioned here because an increasing number of reports indicate that viruses themselves can hijack these processes to their advantage to modulate the metabolism of their own RNAs, in addition to negative impact cellular defenses [89, 90, 91]. It is for the moment unclear whether the activity of ISG20 can be altered by the variety or the abundance of specific epitranscriptomic marks, because exhaustive studies of all these modifications in the context of specific viruses are lacking (Table 1).

However, we can hypothesize that the extent and or abundance of epitranscriptomic marks in a given RNA could modulate either directly or indirectly through RNA‐binding proteins the action of ISG20. The weight of these modifications in the antiviral functions of ISG20 can be direct, for instance, by providing protection from direct viral RNA degradation, or indirect, for example, by altering the efficiency of translation by specialized subsets of ribosomes, as proposed in the translation inhibition models.

## Concluding remarks

Overall, several studies indicate that ISG20 is an important and broad antiviral effector during innate immune responses. While the exonuclease activity of ISG20 is deemed necessary for its antiviral activity toward multiple viruses, the identity of the nucleic acids targeted by ISG20 remains unclear, ranging from viral RNA to deaminated single‐stranded DNA or to cellular RNA targets that may influence translation. Rarely mentioned, high levels of ISG20 expression lead to cell death [39, 92] and it is at present unknown whether regulatory mechanisms exist that keep in check this deleterious effect. In addition to highlighting the importance of carefully controlling ISG20 expression levels during viral replication studies, these results indicate that the specificity of ISG20 for its viral targets is not absolute. Indeed, under certain circumstances the cell can also be susceptible to ISG20, stressing all the more the need to identify the RNA features that govern susceptibility to this factor.

Despite the fact that the underlying molecular mechanism leading to viral inhibition remains to be defined precisely, ISG20 has shown potential for use as broad antiviral inhibitor after vehiculation inside the cell as fusion protein with antibodies (Fc fragment) or with Tat cell‐penetrating peptides [93, 94]. In addition, ISG20 expression has been also associated with glioma and radioresistance in oral cancer cells and other types of cancer [18, 48, 95]. As such, the molecular definition of its mechanism of action in the cell could spur novel developments in antiviral treatment and yield clues to other cellular processes of broader interest. Future studies will certainly shed new light on several key aspects of the biology of ISG20.

## Conflict of interest

The authors have declared that there is no conflict of interest.

## Author contribution

SD, CL, FF, and AC conceived and wrote the manuscript.

## References

[1] Isaacs A , Lindenmann J . Virus interference: I. The interferon. By Alick Isaacs and Jean Lindenmann, 1957. CA Cancer J Clin. 1957;38:280–90. 2458172

[2] Schoggins JW . Interferon‐stimulated genes: what do they all do? Annu Rev Virol. 2019;6:567–84. 31283436 10.1146/annurev-virology-092818-015756

[3] McNab F , Mayer‐Barber K , Sher A , Wack A , O’Garra A . Type I interferons in infectious disease. Nat Rev Immunol. 2015;15:87–103. 25614319 10.1038/nri3787 PMC7162685

[4] Manry J , Laval G , Patin E , Fornarino S , Itan Y , Fumagalli M , et al. Evolutionary genetic dissection of human interferons. J Exp Med. 2011;208:2747–59. 22162829 10.1084/jem.20111680 PMC3244034

[5] Capobianchi MR , Uleri E , Caglioti C , Dolei A . Type I IFN family members: Similarity, differences and interaction. Cytokine Growth Factor Rev. 2015;26:103–11. 25466633 10.1016/j.cytogfr.2014.10.011 PMC7108279

[6] Sadler AJ , Williams BRG . Interferon‐inducible antiviral effectors. Nat Rev Immunol. 2008;8:559–68. 18575461 10.1038/nri2314 PMC2522268

[7] Gongora C , David G , Pintard L , Tissot C , Hua TD , Dejean A , et al. Molecular cloning of a new interferon‐induced PML nuclear body‐associated protein. J Biol Chem. 1997;272:19457–63. 9235947 10.1074/jbc.272.31.19457

[8] Pentecost BT . Expression and estrogen regulation of the HEM45 mRNA in human tumor lines and in the rat uterus. J Steroid Biochem Mol Biol. 1998;64:25–33. 9569007 10.1016/s0960-0760(97)00140-4

[9] Li X‐L , Blackford JA , Hassel BA . RNase L Mediates the Antiviral Effect of Interferon through a Selective Reduction in Viral RNA during Encephalomyocarditis Virus Infection. J Virol. 1998;72:2752–9. 9525594 10.1128/jvi.72.4.2752-2759.1998 PMC109719

[10] Nguyen LH , Espert L , Mechti N , Wilson DM . The human interferon‐ and estrogen‐regulated *ISG20/HEM45* Gene product degrades single‐stranded RNA and DNA in Vitro ^†^ . Biochemistry. 2001;40:7174–9. 11401564 10.1021/bi010141t

[11] Gongora C , Degols G , Espert L , Hua TD , Mechti N . A unique ISRE, in the TATA‐less human Isg20 promoter, confers IRF‐1‐mediated responsiveness to both interferon type I and type II. Nucleic Acids Res. 2000;28:2333–41. 10871365 10.1093/nar/28.12.2333 PMC102735

[12] Weiss CM , Trobaugh DW , Sun C , Lucas TM , Diamond MS , Ryman KD , et al. The interferon‐induced exonuclease ISG20 exerts antiviral activity through upregulation of type I interferon response proteins. mSphere. 2018;3:18. 10.1128/mSphere.00209-18 PMC6147134 30232164

[13] Imaizumi T , Mechti N , Matsumiya T , Sakaki H , Kubota K , Yoshida H , et al. Expression of interferon‐stimulated gene 20 in vascular endothelial cells. Microbiol Immunol. 2008;52:30–5. 18352910 10.1111/j.1348-0421.2008.00004.x

[14] Wang J , Wang Y , Xing P , Liu Q , Zhang C , Sui Y , et al. Development and validation of a hypoxia‐related prognostic signature for breast cancer. Oncol Lett. 2020;20:1906–14. 32724434 10.3892/ol.2020.11733 PMC7377061

[15] Alsheikh HAM , Metge BJ , Pruitt HC , Kammerud SC , Chen D , Wei S , et al. Disruption of STAT5A and NMI signaling axis leads to ISG20‐driven metastatic mammary tumors. Oncogenesis. 2021;10:45. 34078871 10.1038/s41389-021-00333-y PMC8172570

[16] Qu X , Shi Z , Guo J , Guo C , Qiu J , Hua K . Identification of a novel six‐gene signature with potential prognostic and therapeutic value in cervical cancer. Cancer Med. 2021;10:6881–96. 34498424 10.1002/cam4.4054 PMC8495282

[17] Yu J , Liu T‐T , Liang L‐L , Liu J , Cai H‐Q , Zeng J , et al. Identification and validation of a novel glycolysis‐related gene signature for predicting the prognosis in ovarian cancer. Cancer Cell Int. 2021;21:353. 34229669 10.1186/s12935-021-02045-0 PMC8258938

[18] Miyashita H , Fukumoto M , Kuwahara Y , Takahashi T , Fukumoto M . ISG20 is overexpressed in clinically relevant radioresistant oral cancer cells. Int J Clin Exp Pathol. 2020;13:1633–9. 32782682 PMC7414473

[19] Xu T , Ruan H , Gao S , Liu J , Liu Y , Song Z , et al. ISG20 serves as a potential biomarker and drives tumor progression in clear cell renal cell carcinoma. Aging. 2020;12:1808–27. 32003757 10.18632/aging.102714 PMC7053611

[20] Lin S‐L , Wu S‐M , Chung I‐H , Lin Y‐H , Chen C‐Y , Chi H‐C , et al. Stimulation of interferon‐stimulated gene 20 by thyroid hormone enhances angiogenesis in liver cancer. Neoplasia. 2018;20:57–68. 29195126 10.1016/j.neo.2017.10.007 PMC5721268

[21] Van Tong H , Hoan NX , Binh MT , Quyen DT , Meyer CG , Song LH , et al. Interferon‐stimulated gene 20 kDa protein serum levels and clinical outcome of hepatitis B virus‐related liver diseases. Oncotarget. 2018;9:27858–71. 29963243 10.18632/oncotarget.25559 PMC6021248

[22] Lee J‐H , Koh YA , Cho C‐K , Lee S‐J , Lee Y‐S , Bae S . Identification of a novel ionizing radiation‐induced nuclease, AEN, and its functional characterization in apoptosis. Biochem Biophys Res Comm. 2005;337:39–47. 16171785 10.1016/j.bbrc.2005.08.264

[23] Couté Y , Kindbeiter K , Belin S , Dieckmann R , Duret L , Bezin L , et al. ISG20L2, a novel vertebrate nucleolar exoribonuclease involved in ribosome biogenesis. Mol Cell Proteomics. 2008;7:546–59. 18065403 10.1074/mcp.M700510-MCP200

[24] Zhou Z , Wang N , Woodson SE , Dong Q , Wang J , Liang Y , et al. Antiviral activities of ISG20 in positive‐strand RNA virus infections. Virology. 2011;409:175–88. 21036379 10.1016/j.virol.2010.10.008 PMC3018280

[25] Shevelev IV , Hübscher U . The 3′–5′ exonucleases. Nat Rev Mol Cell Biol. 2002;3:364–76. 11988770 10.1038/nrm804

[26] Mason PA , Cox LS . The role of DNA exonucleases in protecting genome stability and their impact on ageing. AGE. 2012;34:1317–40. 21948156 10.1007/s11357-011-9306-5 PMC3528374

[27] Zuo Y . Exoribonuclease superfamilies: structural analysis and phylogenetic distribution. Nucleic Acids Res. 2001;29:1017–26. 11222749 10.1093/nar/29.5.1017 PMC56904

[28] Viswanathan M , Lovett ST . Exonuclease X of Escherichia coli. J Biol Chem. 1999;274:30094–100. 10514496 10.1074/jbc.274.42.30094

[29] Horio T , Murai M , Inoue T , Hamasaki T , Tanaka T , Ohgi T . Crystal structure of human ISG20, an interferon‐induced antiviral ribonuclease. FEBS Lett. 2004;577:111–6. 15527770 10.1016/j.febslet.2004.09.074

[30] Liu Y , Nie H , Mao R , Mitra B , Cai D , Yan R , et al. Interferon‐inducible ribonuclease ISG20 inhibits hepatitis B virus replication through directly binding to the epsilon stem‐loop structure of viral RNA. PLoS Pathog. 2017;13:e1006296. 28399146 10.1371/journal.ppat.1006296 PMC5388505

[31] Beck J . Hepatitis B virus replication. WJG. 2007;13:48. 17206754 10.3748/wjg.v13.i1.48 PMC4065876

[32] Kramvis A , Kew MC . Structure and function of the encapsidation signal of hepadnaviridae. J Viral Hepat. 1998;5:357–67. 9857345 10.1046/j.1365-2893.1998.00124.x

[33] Flodell S . The apical stem‐loop of the hepatitis B virus encapsidation signal folds into a stable tri‐loop with two underlying pyrimidine bulges. Nucleic Acids Res. 2002;30:4803–11. 12409471 10.1093/nar/gkf603 PMC135823

[34] LeBlanc RM , Kasprzak WK , Longhini AP , Olenginski LT , Abulwerdi F , Ginocchio S , et al. Structural insights of the conserved “priming loop” of hepatitis B virus pre‐genomic RNA. J Biomol Struct Dyn. 2021;44:1–13. 10.1080/07391102.2021.1934544 PMC10167916 34155954

[35] Stadler D , Kächele M , Jones AN , Hess J , Urban C , Schneider J , et al. (Interferon‐induced degradation of the persistent hepatitis B virus cccDNA form depends on ISG20. EMBO Rep. 2021;22:68. 10.15252/embr.201949568 PMC8183418 33969602

[36] Espert L , Degols G , Lin Y‐L , Vincent T , Benkirane M , Mechti N . Interferon‐induced exonuclease ISG20 exhibits an antiviral activity against human immunodeficiency virus type 1. J Gen Virol. 2005;86:2221–9. 16033969 10.1099/vir.0.81074-0

[37] Espert L , Degols G , Gongora C , Blondel D , Williams BR , Silverman RH , et al. ISG20, a New interferon‐induced rnase specific for single‐stranded RNA, defines an alternative antiviral pathway against RNA genomic viruses. J Biol Chem. 2003;278:16151–8. 12594219 10.1074/jbc.M209628200

[38] Jiang D , Weidner JM , Qing M , Pan X‐B , Guo H , Xu C , et al. Identification of five interferon‐induced cellular proteins that inhibit west nile virus and dengue virus infections. J Virol. 2010;84:8332–41. 20534863 10.1128/JVI.02199-09 PMC2916517

[39] Wu N , Nguyen X‐N , Wang L , Appourchaux R , Zhang C , Panthu B , et al. The interferon stimulated gene 20 protein (ISG20) is an innate defense antiviral factor that discriminates self versus non‐self translation. PLoS Pathog. 2019;15:e1008093. 31600344 10.1371/journal.ppat.1008093 PMC6805002

[40] Hu J , Seeger C . Hepadnavirus genome replication and persistence. Cold Spring Harb Perspect Med. 2015;5:a021386. 26134841 10.1101/cshperspect.a021386 PMC4484952

[41] Tsukuda S , Watashi K . Hepatitis B virus biology and life cycle. Antiviral Res. 2020;182:104925. 32866519 10.1016/j.antiviral.2020.104925

[42] Gaudray G , Gachon F , Basbous J , Biard‐Piechaczyk M , Devaux C , Mesnard J‐M . The Complementary Strand of the Human T‐Cell Leukemia Virus Type 1 RNA Genome Encodes a bZIP Transcription Factor That Down‐Regulates Viral Transcription. J Virol. 2002;76:12813–22. 12438606 10.1128/JVI.76.24.12813-12822.2002 PMC136662

[43] Savoret J , Mesnard J‐M , Gross A , Chazal N . Antisense transcripts and antisense protein: a new perspective on human immunodeficiency virus type 1. Front Microbiol. 2021;11:625941. 33510738 10.3389/fmicb.2020.625941 PMC7835632

[44] Blondel D , Maarifi G , Nisole S , Chelbi‐Alix M . Resistance to rhabdoviridae infection and subversion of antiviral responses. Viruses. 2015;7:3675–702. 26198243 10.3390/v7072794 PMC4517123

[45] Guu TSY , Zheng W , Tao YJ . Bunyavirus: Structure and Replication. In: Rossmann MG , Rao VB , editors. Viral Molecular Machines. US, Boston, MA: Springer; 2012. p. 245–66.

[46] Sun X , Li D , Wang Z , Liu Q , Wei Y , Liu T . A dimorphism shift of hepatitis B virus capsids in response to ionic conditions. Nanoscale. 2018;10:16984–9. 30183040 10.1039/c8nr03370f

[47] Couch RB . Orthomyxoviruses. In: Baron S , editors. Medical Microbiology. 4th ed. Galveston, TX: University of Texas Medical Branch at Galveston; 1996. 21413353

[48] Zheng Z , Wang L , Pan J . Interferon‐stimulated gene 20‐kDa protein (ISG20) in infection and disease: Review and outlook. Intractable Rare Dis Res. 2017;6:35–40. 28357179 10.5582/irdr.2017.01004 PMC5359350

[49] Leong CR , Funami K , Oshiumi H , Mengao D , Takaki H , Matsumoto M , et al. Interferon‐stimulated gene of 20 kDa protein (ISG20) degrades RNA of hepatitis B virus to impede the replication of HBV *in vitro* and *in vivo* . Oncotarget. 2016;7:68179–93. 27626689 10.18632/oncotarget.11907 PMC5356548

[50] Imam H , Khan M , Gokhale NS , McIntyre ABR , Kim G‐W , Jang JY , et al. *N6* ‐methyladenosine modification of hepatitis B virus RNA differentially regulates the viral life cycle. Proc Natl Acad Sci USA. 2018;115:8829–34. 30104368 10.1073/pnas.1808319115 PMC6126736

[51] Shi H , Wei J , He C . Where, When, and How: Context‐Dependent Functions of RNA Methylation Writers, Readers, and Erasers. Mol Cell. 2019;74:640–50. 31100245 10.1016/j.molcel.2019.04.025 PMC6527355

[52] Malim MH . APOBEC proteins and intrinsic resistance to HIV‐1 infection. Phil Trans R Soc B. 2009;364:675–87. 19038776 10.1098/rstb.2008.0185 PMC2660912

[53] Harris RS , Dudley JP . APOBECs and virus restriction. Virology. 2015;479–480:131–45. 10.1016/j.virol.2015.03.012 PMC4424171 25818029

[54] Vartanian J‐P , Sommer P , Wain‐Hobson S . Death and the retrovirus. Trends Mol Med. 2003;9:409–13. 14557052 10.1016/j.molmed.2003.08.008

[55] Stenglein MD , Burns MB , Li M , Lengyel J , Harris RS . APOBEC3 proteins mediate the clearance of foreign DNA from human cells. Nat Struct Mol Biol. 2010;17:222–9. 20062055 10.1038/nsmb.1744 PMC2921484

[56] Bohn M‐F , Shandilya SMD , Silvas TV , Nalivaika EA , Kouno T , Kelch BA , et al. The ssDNA Mutator APOBEC3A is regulated by cooperative dimerization. Structure. 2015;23:903–11. 25914058 10.1016/j.str.2015.03.016 PMC4874493

[57] Hoopes JI , Cortez LM , Mertz TM , Malc EP , Mieczkowski PA , Roberts SA . APOBEC3A and APOBEC3B preferentially deaminate the lagging strand template during DNA replication. Cell Rep. 2016;14:1273–82. 26832400 10.1016/j.celrep.2016.01.021 PMC4758883

[58] Berger G , Durand S , Fargier G , Nguyen X‐N , Cordeil S , Bouaziz S , et al. APOBEC3A Is a Specific Inhibitor of the Early Phases of HIV‐1 Infection in Myeloid Cells. PLoS Pathog. 2011;7:e1002221. 21966267 10.1371/journal.ppat.1002221 PMC3178557

[59] Duncan BK , Miller JH . Mutagenic deamination of cytosine residues in DNA. Nature. 1980;287:560–1. 6999365 10.1038/287560a0

[60] Ehrlich M , Zhang X‐Y , Inamdar NM . Spontaneous deamination of cytosine and 5‐methylcytosine residues in DNA and replacement of 5‐methylcytosine residues with cytosine residues. Mutation Res/Rev Genetic Toxicol. 1990;238:277–86. 10.1016/0165-1110(90)90019-8 2188124

[61] Diamond MS , Farzan M . The broad‐spectrum antiviral functions of IFIT and IFITM proteins. Nat Rev Immunol. 2013;13:46–57. 23237964 10.1038/nri3344 PMC3773942

[62] Diamond MS . IFIT1: A dual sensor and effector molecule that detects non‐2′‐O methylated viral RNA and inhibits its translation. Cytokine Growth Factor Rev. 2014;25:543–50. 24909568 10.1016/j.cytogfr.2014.05.002 PMC4234691

[63] Thompson SR , Gulyas KD , Sarnow P . Internal initiation in Saccharomyces cerevisiae mediated by an initiator tRNA/eIF2‐independent internal ribosome entry site element. Proc Natl Acad Sci. 2001;98:12972–7. 11687653 10.1073/pnas.241286698 PMC60809

[64] Eulalio A , Behm‐Ansmant I , Izaurralde E . P bodies: at the crossroads of post‐transcriptional pathways. Nat Rev Mol Cell Biol. 2007;8:9–22. 17183357 10.1038/nrm2080

[65] Rodriguez‐Corona U , Sobol M , Rodriguez‐Zapata LC , Hozak P , Castano E . Fibrillarin from Archaea to human: Review on fibrillarin. Biol Cell. 2015;107:159–74. 25772805 10.1111/boc.201400077

[66] Dimitrova DG , Teysset L , Carré C . RNA 2’‐O‐Methylation (Nm) Modification in Human Diseases. Genes. 2019;10:117. 10.3390/genes10020117 PMC6409641 30764532

[67] Genuth NR , Barna M . The discovery of ribosome heterogeneity and its implications for gene regulation and organismal life. Mol Cell. 2018;71:364–74. 30075139 10.1016/j.molcel.2018.07.018 PMC6092941

[68] Monaco P , Marcel V , Diaz J‐J , Catez F . 2′‐O‐Methylation of Ribosomal RNA: Towards an Epitranscriptomic Control of Translation? Biomolecules. 2018;8:106. 10.3390/biom8040106 PMC6316387 30282949

[69] Ferretti MB , Karbstein K . Does functional specialization of ribosomes really exist? RNA. 2019;25:521–38. 30733326 10.1261/rna.069823.118 PMC6467006

[70] Espert L , Eldin P , Gongora C , Bayard B , Harper F , Chelbi‐Alix MK , et al. The exonuclease ISG20 mainly localizes in the nucleolus and the Cajal (Coiled) bodies and is associated with nuclear SMN protein‐containing complexes. J Cell Biochem. 2006;98:1320–33. 16514659 10.1002/jcb.20869

[71] Kelly C , Van Driel R , Wilkinson GWG . Disruption of PML‐associated nuclear bodies during human cytomegalovirus infection. J Gen Virol. 1995;76:2887–93. 7595400 10.1099/0022-1317-76-11-2887

[72] Regad T , Chelbi‐Alix MK . Role and fate of PML nuclear bodies in response to interferon and viral infections. Oncogene. 2001;20:7274–86. 11704856 10.1038/sj.onc.1204854

[73] Everett RD , Chelbi‐Alix MK . PML and PML nuclear bodies: Implications in antiviral defence. Biochimie. 2007;89:819–30. 17343971 10.1016/j.biochi.2007.01.004

[74] Bernardi R , Pandolfi PP . Structure, dynamics and functions of promyelocytic leukaemia nuclear bodies. Nat Rev Mol Cell Biol. 2007;8:1006–16. 17928811 10.1038/nrm2277

[75] Dundr M , Misteli T . Biogenesis of Nuclear Bodies. Cold Spring Harbor Perspect Biol. 2010;2:a000711. 10.1101/cshperspect.a000711 PMC2982170 21068152

[76] Mao YS , Zhang B , Spector DL . Biogenesis and function of nuclear bodies. Trends Genet. 2011;27:295–306. 21680045 10.1016/j.tig.2011.05.006 PMC3144265

[77] Courchaine EM , Lu A , Neugebauer KM . Droplet organelles? EMBO J. 2016;35:1603–12. 27357569 10.15252/embj.201593517 PMC4969579

[78] Trinkle‐Mulcahy L , Sleeman JE . The Cajal body and the nucleolus: “In a relationship” or “It's complicated”? RNA Biol. 2017;14:739–51. 10.1080/15476286.2016.1236169 27661468 PMC5519233

[79] Decker CJ , Parker R . P‐bodies and stress granules: possible roles in the control of translation and mRNA degradation. Cold Spring Harbor Perspect Biol. 2012;4:a012286. 10.1101/cshperspect.a012286 PMC3428773 22763747

[80] Feng J , Wickenhagen A , Turnbull ML , Rezelj VV , Kreher F , Tilston‐Lunel NL , et al. Interferon‐Stimulated Gene (ISG)‐Expression Screening Reveals the Specific Antibunyaviral Activity of ISG20. J Virol. 2018;92:e02140–17. 29695422 10.1128/JVI.02140-17 PMC6002717

[81] Fu Y , Dominissini D , Rechavi G , He C . Gene expression regulation mediated through reversible m 6 A RNA methylation. Nat Rev Genet. 2014;15:293–306. 24662220 10.1038/nrg3724

[82] Frye M , Harada BT , Behm M , He C . RNA modifications modulate gene expression during development. Science. 2018;361:1346–9. 30262497 10.1126/science.aau1646 PMC6436390

[83] Yang Y , Hsu PJ , Chen Y‐S , Yang Y‐G . Dynamic transcriptomic m6A decoration: writers, erasers, readers and functions in RNA metabolism. Cell Res. 2018;28:616–24. 10.1038/s41422-018-0040-8 29789545 PMC5993786

[84] Wiener D , Schwartz S . The epitranscriptome beyond m6A. Nat Rev Genet. 2021;22:119–31. 33188361 10.1038/s41576-020-00295-8

[85] Wang X , He C . Dynamic RNA Modifications in Posttranscriptional Regulation. Mol Cell. 2014;56:5–12. 25280100 10.1016/j.molcel.2014.09.001 PMC7129666

[86] Kan RL , Chen J , Sallam T . Crosstalk between epitranscriptomic and epigenetic mechanisms in gene regulation. Trends Genet. 2022;38:182–93. 10.1016/j.tig.2021.06.014 34294427 PMC9093201

[87] Harcourt EM , Kietrys AM , Kool ET . Chemical and structural effects of base modifications in messenger RNA. Nature. 2017;541:339–46. 28102265 10.1038/nature21351 PMC5498787

[88] Boo SH , Kim YK . The emerging role of RNA modifications in the regulation of mRNA stability. Exp Mol Med. 2020;52:400–8. 32210357 10.1038/s12276-020-0407-z PMC7156397

[89] Netzband R , Pager CT . Epitranscriptomic marks: Emerging modulators of RNA virus gene expression. WIREs RNA. 2020;11. 10.1002/wrna.1576 PMC7169815 31694072

[90] Daffis S , Szretter KJ , Schriewer J , Li J , Youn S , Errett J , et al. 2′‐O methylation of the viral mRNA cap evades host restriction by IFIT family members. Nature. 2010;468:452–6. 21085181 10.1038/nature09489 PMC3058805

[91] Tsai K , Cullen BR . Epigenetic and epitranscriptomic regulation of viral replication. Nat Rev Microbiol. 2020;18:559–70. 32533130 10.1038/s41579-020-0382-3 PMC7291935

[92] Degols G , Eldin P , Mechti N . ISG20, an actor of the innate immune response. Biochimie. 2007;89:831–5. 17445960 10.1016/j.biochi.2007.03.006

[93] Ding J , Aldo P , Roberts CM , Stabach P , Liu H , You Y , et al. Placenta‐derived interferon‐stimulated gene 20 controls ZIKA virus infection. EMBO Rep. 2021;22:2450. 10.15252/embr.202152450 PMC8490983 34405956

[94] Liu K , Li Y , Zhou B , Wang F , Huan B , Shao D , et al. A conjugate protein containing HIV TAT, ISG20, and a PRRSV polymerase binding inhibits PRRSV replication and may be a novel therapeutic platform. Res Vet Sci. 2017;113:13–20. 28818749 10.1016/j.rvsc.2017.08.008

[95] Gao M , Lin Y , Liu X , Li Y , Zhang C , Wang Z , et al. ISG20 promotes local tumor immunity and contributes to poor survival in human glioma. OncoImmunology. 2019;8:e1534038. 30713788 10.1080/2162402X.2018.1534038 PMC6343791

[96] Qu H , Li J , Yang L , Sun L , Liu W , He H . Influenza A Virus‐induced expression of ISG20 inhibits viral replication by interacting with nucleoprotein. Virus Genes. 2016;52:759–67. 27342813 10.1007/s11262-016-1366-2

[97] Krug RM , Morgan MA , Shatkin AJ . Influenza viral mRNA contains internal N6‐methyladenosine and 5’‐terminal 7‐methylguanosine in cap structures. J Virol. 1976;20:45–53. 1086370 10.1128/jvi.20.1.45-53.1976 PMC354964

[98] Courtney DG , Kennedy EM , Dumm RE , Bogerd HP , Tsai K , Heaton NS , et al. Epitranscriptomic Enhancement of Influenza A Virus Gene Expression and Replication. Cell Host Microbe. 2017;22:377–386.e5. 28910636 10.1016/j.chom.2017.08.004 PMC5615858

[99] Olschewski S , Cusack S , Rosenthal M . The Cap‐Snatching Mechanism of Bunyaviruses. Trends Microbiol. 2020;28:293–303. 31948728 10.1016/j.tim.2019.12.006

[100] Dunn EF , Pritlove DC , Elliott RM . The S RNA genome segments of Batai, Cache Valley, Guaroa, Kairi, Lumbo, Main Drain and Northway bunyaviruses: sequence determination and analysis. J Gen Virol. 1994;75:597–608. 8126455 10.1099/0022-1317-75-3-597

[101] Wang JT , McElvain LE , Whelan SPJ . Vesicular Stomatitis Virus mRNA Capping Machinery Requires Specific *cis* ‐Acting Signals in the RNA. J Virol. 2007;81:11499–506. 17686869 10.1128/JVI.01057-07 PMC2045530

[102] Qiu W , Zhang Q , Zhang R , Lu Y , Wang X , Tian H , et al. N6‐methyladenosine RNA modification suppresses antiviral innate sensing pathways via reshaping double‐stranded RNA. Nat Commun. 2021;12:1582. 33707441 10.1038/s41467-021-21904-y PMC7952553

[103] Hwang LN , Englund N , Pattnaik AK . Polyadenylation of Vesicular Stomatitis Virus mRNA Dictates Efficient Transcription Termination at the Intercistronic Gene Junctions. J Virol. 1998;72:1805–13. 9499031 10.1128/jvi.72.3.1805-1813.1998 PMC109470

[104] Jiang D , Guo H , Xu C , Chang J , Gu B , Wang L , et al. Identification of three interferon‐inducible cellular enzymes that inhibit the replication of hepatitis C virus. J Virol. 2008;82:1665–78. 18077728 10.1128/JVI.02113-07 PMC2258705

[105] Jia Y , Wei L , Jiang D , Cong X , Fei R . Investigating the inhibitory effects of interferon‐alpha on the replication of hepatitis C virus replicon. Zhonghua Yi Xue Za Zhi. 2005;85:2065–9. 16313804

[106] Bradrick SS . The hepatitis C virus 3’‐untranslated region or a poly(A) tract promote efficient translation subsequent to the initiation phase. Nucleic Acids Res. 2006;34:1293–303. 16510853 10.1093/nar/gkl019 PMC1388098

[107] Moes L , Wirth M . The internal initiation of translation in bovine viral diarrhea virus RNA depends on the presence of an RNA pseudoknot upstream of the initiation codon. Virol J. 2007;4:124. 18034871 10.1186/1743-422X-4-124 PMC2212637

[108] McIntyre W , Netzband R , Bonenfant G , Biegel JM , Miller C , Fuchs G , et al. Positive‐sense RNA viruses reveal the complexity and dynamics of the cellular and viral epitranscriptomes during infection. Nucleic Acids Res. 2018;46:5776–91. 29373715 10.1093/nar/gky029 PMC6009648

[109] Kolykhalov AA , Feinstone SM , Rice CM . Identification of a highly conserved sequence element at the 3’ terminus of hepatitis C virus genome RNA. J Virol. 1996;70:3363–71. 8648666 10.1128/jvi.70.6.3363-3371.1996 PMC190207

[110] Collett MS , Anderson DK , Retzel E . Comparisons of the pestivirus bovine viral diarrhoea virus with members of the flaviviridae. J Gen Virol. 1988;69:2637–43. 2844971 10.1099/0022-1317-69-10-2637

[111] Zhou Y , Ray D , Zhao Y , Dong H , Ren S , Li Z , et al. Structure and Function of Flavivirus NS5 Methyltransferase. J Virol. 2007;81:3891–903. 17267492 10.1128/JVI.02704-06 PMC1866096

[112] Dong H , Ren S , Zhang B , Zhou Y , Puig‐Basagoiti F , Li H , et al. West nile virus methyltransferase catalyzes two methylations of the viral RNA cap through a substrate‐repositioning mechanism. J Virol. 2008;82:4295–307. 18305027 10.1128/JVI.02202-07 PMC2293060

[113] Saeedi BJ , Geiss BJ . Regulation of flavivirus RNA synthesis and capping: Regulation of flavivirus RNA synthesis and capping. Wires RNA. 2013;4:723–35. 23929625 10.1002/wrna.1191 PMC3797245

[114] Song Y , Mugavero J , Stauft CB , Wimmer E . Dengue and Zika Virus 5′ Untranslated Regions Harbor Internal Ribosomal Entry Site Functions. MBio. 2019;10:19. 10.1128/mBio.00459-19 PMC6456755 30967466

[115] Gokhale NS , McIntyre ABR , McFadden MJ , Roder AE , Kennedy EM , Gandara JA , et al. N6 ‐methyladenosine in flaviviridae viral RNA genomes regulates infection. Cell Host & Microbe. 2016;20:654–65. 10.1016/j.chom.2016.09.015 27773535 PMC5123813

[116] Bergamini G , Preiss T , Hentze MW . Picornavirus IRESes and the poly(A) tail jointly promote cap‐independent translation in a mammalian cell‐free system. RNA. 2000;6:1781–90. 11142378 10.1017/s1355838200001679 PMC1370048

[117] Jang SK , Pestova TV , Hellen CUT , Witherell GW , Wimmer E . Cap‐Independent Translation of Picornavirus RN As: Structure and Function of the Internal Ribosomal Entry Site. Enzyme. 1990;44:292–309. 1966843 10.1159/000468766

[118] Decroly E , Ferron F , Lescar J , Canard B . Conventional and unconventional mechanisms for capping viral mRNA. Nat Rev Microbiol. 2012;10:51–65. 10.1038/nrmicro2675 PMC7097100 22138959

[119] Zhang Y , Burke CW , Ryman KD , Klimstra WB . Identification and characterization of interferon‐induced proteins that inhibit alphavirus replication. J Virol. 2007;81:11246–55. 17686841 10.1128/JVI.01282-07 PMC2045553

[120] Hyde JL , Diamond MS . Innate immune restriction and antagonism of viral RNA lacking 2׳‐O methylation. Virology. 2015;479–480:66–74. 10.1016/j.virol.2015.01.019 PMC4424151 25682435

[121] Hsuchen C‐C , Dubin DT . Di‐ and trimethylated congeners of 7‐methylguanine in Sindbis virus mRNA. Nature. 1976;264:190–1. 995206 10.1038/264190a0

[122] Furutani Y , Toguchi M , Higuchi S , Yanaka K , Gailhouste L , Qin X‐Y , et al. Establishment of a Rapid Detection System for ISG20‐Dependent SARS‐CoV‐2 Subreplicon RNA Degradation Induced by Interferon‐α. IJMS. 2021;22:11641. 34769072 10.3390/ijms222111641 PMC8583800

[123] Nakagawa K , Lokugamage KG , Makino S . Viral and Cellular mRNA Translation in Coronavirus‐Infected Cells. Advances in Virus Research. Amsterdam: Elsevier; 2016. p. 165–92. 10.1016/bs.aivir.2016.08.001 PMC5388242 27712623

[124] Kim D , Lee J‐Y , Yang J‐S , Kim JW , Kim VN , Chang H . The Architecture of SARS‐CoV‐2 Transcriptome. Cell. 2020;181:914–921.e10. 32330414 10.1016/j.cell.2020.04.011 PMC7179501

[125] Liu J , Xu Y‐P , Li K , Ye Q , Zhou H‐Y , Sun H , et al. The m6A methylome of SARS‐CoV‐2 in host cells. Cell Research. 2021;31:404–14. 10.1038/s41422-020-00465-7 33510385 PMC8115241

[126] Zahoor MA , Xue G , Sato H , Murakami T , Takeshima S , Aida Y . HIV‐1 Vpr induces interferon‐stimulated genes in human monocyte‐derived macrophages. PLoS One. 2014;9:e106418. 25170834 10.1371/journal.pone.0106418 PMC4149569

[127] Zahoor MA , Xue G , Sato H , Aida Y . Genome‐wide transcriptional profiling reveals that HIV‐1 Vpr differentially regulates interferon‐stimulated genes in human monocyte‐derived dendritic cells. Virus Res. 2015;208:156–63. 26116899 10.1016/j.virusres.2015.06.017

[128] Wilusz J . Putting an ‘End’ to HIV mRNAs: capping and polyadenylation as potential therapeutic targets. AIDS Res Ther. 2013;10:31. 24330569 10.1186/1742-6405-10-31 PMC3874655

[129] Yedavalli VSRK , Jeang K‐T . Trimethylguanosine capping selectively promotes expression of Rev‐dependent HIV‐1 RNAs. Proc Natl Acad Sci. 2010;107:14787–92. 20679221 10.1073/pnas.1009490107 PMC2930441

[130] Courtney DG . Post‐Transcriptional Regulation of Viral RNA through Epitranscriptional Modification. Cells. 2021;10:1129. 34066974 10.3390/cells10051129 PMC8151693

[131] Doria‐Rose NA , Klein RM , Manion MM , O’Dell S , Phogat A , Chakrabarti B , et al. Frequency and Phenotype of Human Immunodeficiency Virus Envelope‐Specific B Cells from Patients with Broadly Cross‐Neutralizing Antibodies. J Virol. 2009;83:188–99. 18922865 10.1128/JVI.01583-08 PMC2612342

[132] Wieland S , Thimme R , Purcell RH , Chisari FV . Genomic analysis of the host response to hepatitis B virus infection. Proc Natl Acad Sci. 2004;101:6669–74. 15100412 10.1073/pnas.0401771101 PMC404103

[133] Imam H , Kim G‐W , Mir SA , Khan M , Siddiqui A . Interferon‐stimulated gene 20 (ISG20) selectively degrades N6‐methyladenosine modified Hepatitis B Virus transcripts. PLoS Pathog. 2020;16:e1008338. 32059034 10.1371/journal.ppat.1008338 PMC7046284

[134] Seeger C , Mason WS . Molecular biology of hepatitis B virus infection. Virology. 2015;479–480:672–86. 10.1016/j.virol.2015.02.031 PMC4424072 25759099

[135] Block TM , Young JAT , Javanbakht H , Sofia MJ , Zhou T . Host RNA quality control as a hepatitis B antiviral target. Antiviral Res. 2021;186:104972. 33242518 10.1016/j.antiviral.2020.104972

